# Research of deformation law about guide rails under the action of mining deformation in mine vertical shaft

**DOI:** 10.1038/s41598-023-32767-2

**Published:** 2023-04-05

**Authors:** Jianlong Zhao, Chi Ma, Jinna Han, Xingming Xiao, Yuqiang Jiang

**Affiliations:** 1 School of Mechatronic Engineering, China University of Mining and Technology, Xuzhou, 221116 China; 2 School of Chemical Engineering and Technology, China University of Mining and Technology, Xuzhou, 221116 China

**Keywords:** Civil engineering, Mechanical engineering

## Abstract

To lay a foundation for alleviating the influence of mining shaft deformation (MSD) on the guide rail (GR) and monitoring the shaft deformation state, this paper studies the deformation law and mechanism of the guide rail under the MSD. Firstly, a spring is used to simplify the interaction between the shaft lining and surrounding rock soil mass (SRSM) under MSD, and its stiffness coefficient is deduced by the elastic subgrade reaction method. Secondly, a simplified finite element model is established based on spring element, the stiffness coefficient is calculated by the derivation formula, and its effectiveness is verified. Finally, the deformation law and mechanism of GR are analyzed under different types and degrees of MSD, and the deformation characteristics are studied under the disconnection between the shaft, bunton and guide rail. The results show that the established finite element model can better simulate the interaction between the shaft lining and SRSM, and the calculation efficiency is greatly improved. The guide rail deformation (GRD) has a strong ability to characterize MSD and owns the distinctive feature corresponding to different types and degrees of MSD and the connection state. This research can provide reference and guidance for the shaft deformation monitoring and the maintenance and installation of the GR, and also lays a groundwork for studying operation characteristic of hoisting conveyance under MSD.

## Introduction

**Figure 1:**
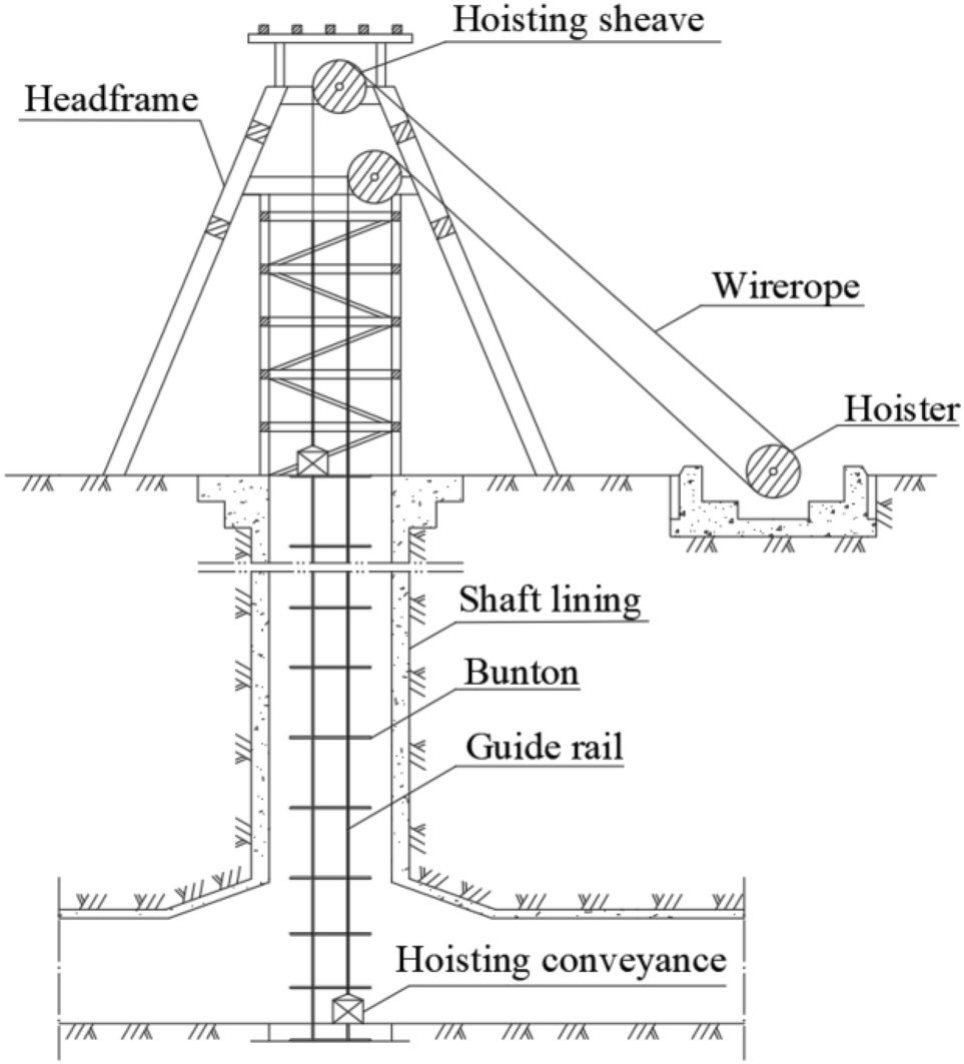
Mine hoisting system.

Mining vertical shaft is the crucial throat engineering, and its deformation state directly determines the safety of coal mine production. Affected by factors such as occurrence of coal seam, lithology, mining method, and unreasonable protective coal pillar, the overlying strata is easily moved and deformed during mining coal seam, which in turn leads to the MSD^1,2^. MSD mainly includes inclination, bending, dislocation, horizontal section change, vertical compression, etc. When the vertical compression occurs, the support effect of the SRSM on the shaft is eliminated in the vertical upward direction, which is different from the force mechanism of other deformation types^3^. MSD not only causes the shaft lining rupture and the water and sand gushing, but also induces the GRD, aggravates the hoisting resistance and instability of the hoisting conveyance, and even leads to jamming or falling^4,5^. A typical mine hoisting system is shown in Fig. 1. Due to the large volume and complex geological conditions of the shaft, its stress and deformation research under the mining action is mainly carried out through field monitoring and numerical calculation. Field monitoring collects ground and shaft deformation data through Global Positioning System, lidar, fiber grating, etc., and analyzes the monitoring data to obtain the shaft deformation law and the main factors leading to its deformation^6,7^.

To deeply understand the main reasons of MSD, the damage mechanism and the influence of construction parameters on its stability, a large number of numerical calculations have been carried out. Kwinta^8^ used a modified Knothe method to predict the continuous displacement of the shaft caused by mining activities. Bruneau^9^ established a shaft numerical analysis model in Map 3D and analyzed the influence of faults and mining sequence on the stability of the main shaft. Sun^5^ employed the Universal Distinct Element Code Trigon method to establish a numerical model of the shaft, and studied its deformation mechanism under backfill mining. Zhao^10^ analyzed the influence of construction parameters on the main shaft stability in Jinchuan No. 3 Mine through a two-dimensional numerical model, mainly including shaft depth, lining thickness and construction technology of releasing displacement. Yan^11^ studied the influence of the backfill compression ratio on the shaft deformation through ABAQUS, and determined the optimal compression ratio for the safety and stability of the shaft. Ma^12^ founded through numerical simulation that the high dip angle of the ore bodies, faults and fractures is the main reason for the collapse of the vertical shaft of Jinchuan Nickel Mine. Dias^13^ used the finite element model established by CESAR-LCPC to analyze the influence of construction sequence and geological deposition on shaft capability, especially induced settlement. Walton^14^ built a three-dimensional finite difference model of circular and elliptical shaft through Universal Distinct Element Code, and studied the factors affecting the relative stability of the shaft geometry. The above research mainly analyzes the influence of geometry, construction parameters and geological conditions on the shaft stability through numerical simulation, dissects the type, law, cause and mechanism of MSD, and neglects its influence on the GRD. The GRD under the MSD not only leads to the change of the operating characteristic in the hoisting system, but also reflects the shaft deformation state to a certain extent, so the GRD law should be studied.

The vertical shaft is a space structure buried deep underground, and the main difference from the above-ground structure is the interaction between the shaft and SRSM. To study the MSD through numerical simulation, it is necessary to establish the model of the shaft lining and SRSM, and its width should be several times the shaft diameter to reduce the influence of boundary effects, which results in excessive calculation scale, so the interaction should be simplified. The interaction between the underground structure and the SRSM is mainly simplified by the spring element. Jeong^15^ established a simplified elastic–plastic foundation beam model by using the *p*–*y* curve spring stiffness coefficient to simplify the interaction between the soil and junction wall, and the calculated results were consistent with the small-scale and full-scale test results. Mitelman^16^ used the spring to simplify the interaction between the ore pillar and body, deduced the estimation equations of its displacement and stress, and verified the accuracy of the simplified method through the finite element model established by Rocscience. Zlatanović^17^ exploited discrete beam-spring elements to study the interaction between soil and tunnel structure, and the comparison with the calculation results of continuous finite element model confirmed its reliability. Ramezani^18^ modeled the foundation soil using torsional and translational springs, calculated the sway and slip modes of the retaining wall, and its result was consistent with those calculated by ANSYS. Sun^19^ simulated pipe–soil interaction through COMBIN39 spring element, and its reliability was confirmed by the theoretical formula. Zhao^20^ used the COMBIN39 spring element to simplify the interaction between the shaft and SRSM under non-mining action, and verified the effectiveness of the simplified method by comparing it with the ANSYS calculation results. The above research shows the feasibility of the spring element to simplify the interaction between the shaft and SRSM under the MSD.

Aiming at the study of GRD law under MSD, it is proposed that a method to simplify the interaction between mining action shaft and SRSM by using spring elements. The calculation formula of the spring stiffness coefficient is deduced by the elastic subgrade reaction method, and a simplified calculation model is established based on the COMBIN39 spring element by ANSYS, and its stiffness coefficient is calculated according to the derivation formula and the effectiveness is verified. By analyzing the GRD laws of different types and degrees of MSD, the interaction mechanism between the shaft and GR is revealed, and the guide deformation characteristic is obtained under different disconnected states between the shaft, bunton and GR.

## Simplification method of the interaction between the shaft lining and surrounding rock soil mass

**Figure 2:**
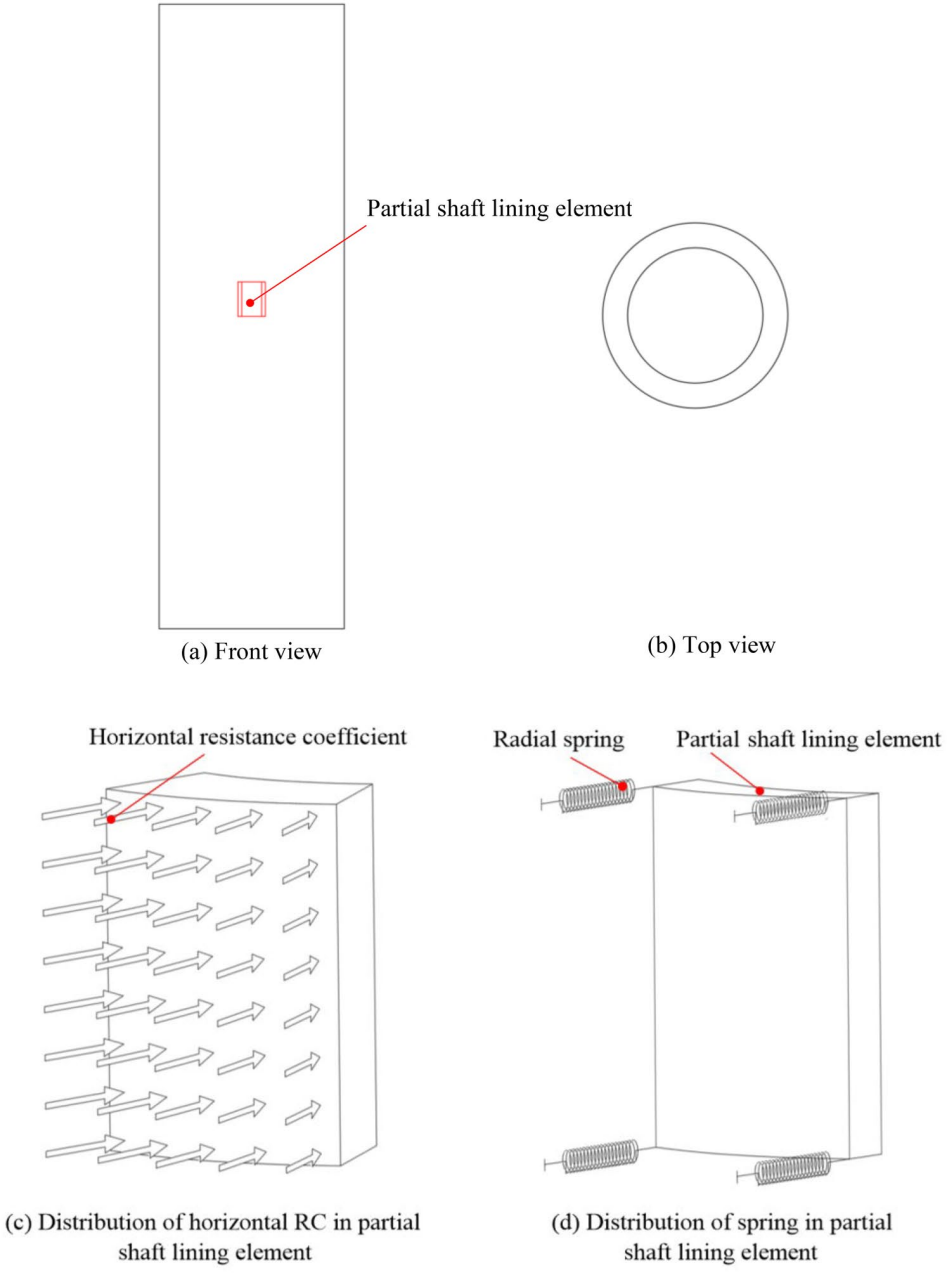
Schematic diagram of the simplification action of SRSM.

Although GRD cannot directly reflect the shaft damage, it can reflect the shaft deformation trend and the mechanism between them to a certain extent, and the feature of the GR is the premise of studying the operating characteristic of the hoisting conveyance under the MSD, hence the study of GRD is of great significance to ensure the safe operation of the hoisting system and shaft. The study of GRD under MSD mainly focuses on the deformation law and characteristic of the GR under various types and degrees of shaft deformation. To facilitate the application of the shaft load, simplify the calculation step and improve the calculation efficiency, the spring element is used to simplify the interaction between the shaft and SRSM. The schematic diagram is shown in Fig. 2. The specific calculation process of the stiffness coefficient of the spring element is as follows.

The elastic subgrade reaction method assumes that the horizontal resistance of the SRSM to the structure is only related to the structural displacement, soil properties and buried depth^21,22^, and the force is proportional to the displacement. Its relationship is shown in Eq. (1).

1 $$ p(z,\;x) = k(z)x^{n} $$

where *p* is the horizontal resistance of the SRSM to structure per unit area, *z* is the depth, *x* is the horizontal displacement of the structure, *k* is the resistance coefficient (RC) in horizontal direction at depth *z*, *n* is the index and $n > 0$, $n = 1$ is the linear elastic subgrade reaction method, $n \ne 1$ is the non-linear elastic subgrade reaction method.

When the rock soil mass around the structure is bedrock, the stability is strong, and it is usually assumed that the RC in the horizontal direction does not change with depth, and is only determined by the lithology^23,24^. When it is soft rock or topsoil, the RC in horizontal direction varies with depth under the same lithology, which is usually determined according to Eq. (2).

2 $$ k(z) = m\;z $$

where *m* is the proportional coefficient of the resistance coefficient (PCRC) in horizontal direction.

Under the same lithology of the bedrock, the RC in horizontal direction does not change with depth, and it is put into Eq. (3) to obtain the spring element stiffness coefficient of the horizontal resistance for the bedrock to the shaft lining.

3 $$ K = \, \pi dhk(z)/L $$

where *d* is the diameter of shaft lining, *h* is the vertical distance between adjacent springs, *L* is the total number of springs in the circumferential direction of the shaft.

**Figure 3:**
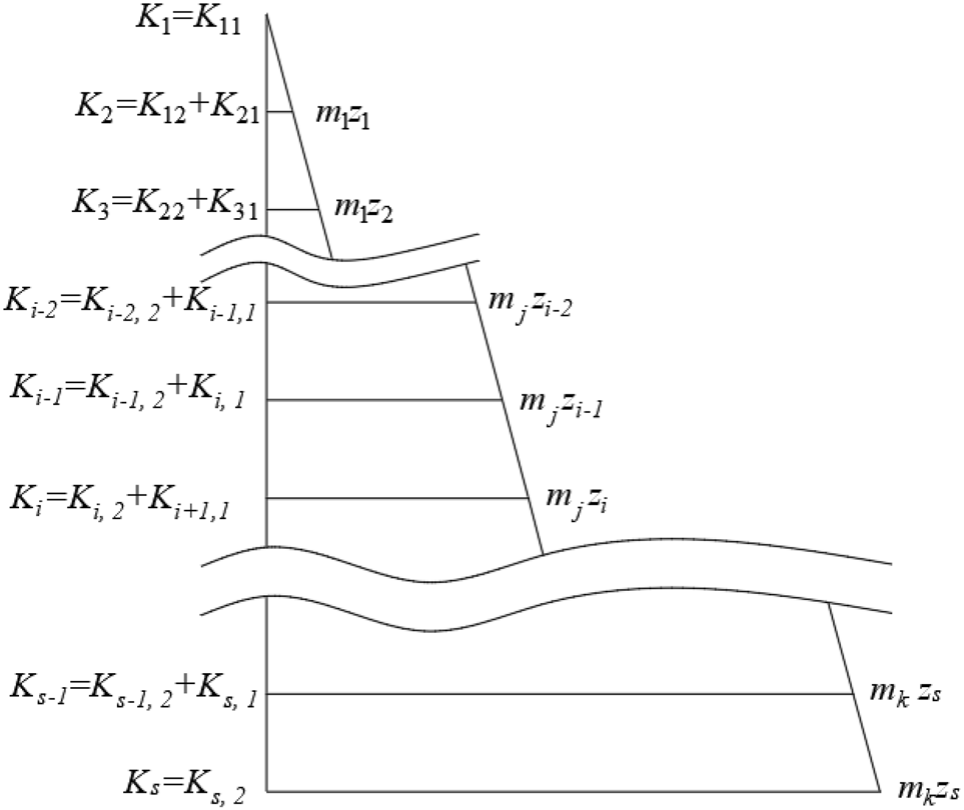
Schematic diagram of spring stiffness coefficients.

On the outer surface of the shaft in the topsoil segment, the total number of layers of springs is *s* from top to bottom. Taking each layer of springs as the dividing line, the shaft is divided into *s* segments. The sum of the scattered horizontal resistance coefficients of the shaft between the first and second springs is calculated. With its middle position as the demarcation point, the sum of the corresponding resistance coefficients on the upper and lower parts is equivalent to the upper and lower springs respectively. The equivalent stiffness coefficient is shown in Eqs. (4) and (5). Schematic diagram of spring stiffness coefficients is shown in Fig. 3.

4 $$ K_{11} = \, \pi dhm_{1} z_{1} /6L $$

5 $$ K_{12} = \, \pi dhm_{1} z_{1} /3L $$

where $m_{1}$ is the PCRC in horizontal direction on the uppermost layer of soil, $z_{1}$ is the depth of the first layer of spring.

In the topsoil segment, the resistance effect of the *i*-th segment shaft is equivalent to the stiffness coefficients of the *i* and *i* + 1 springs as shown in Eqs. (6) and (7). The equivalence principle is the same as in the first part, except that the distribution law of the scattered horizontal resistance coefficients changes.

6 $$ K_{i,1} = \pi m_{j} z_{i} hd/2L + \pi m_{j} (z_{i + 1} - z_{i} )hd/6L = \pi m_{j} hd(z_{i} /3 + z_{i + 1} /6)/L $$

7 $$ K_{i,2} = \pi m_{j} z_{i} hd/2L + \pi m_{j} (z_{i + 1} - z_{i} )hd/3L = \pi m_{j} hd(z_{i} /6 + z_{i + 1} /3)/L $$

where $m_{j}$ is the PCRC in horizontal direction of the *j*-th layer soil, $z_{i}$ and $z_{i + 1}$ are the depths of the springs of the *i* and *i* + 1 layers, respectively.

Combining the above formula, the spring stiffness coefficients of each layer in the topsoil is finally obtained, as shown in Eq. (8).

8 $$ \left\{ {\begin{array}{*{20}l} { \, K_{1} = K_{11} } \hfill \\ { \, K_{i} = K_{i,2} + K_{i + 1,1} \quad \quad 1 < i \le s - 1} \hfill \\ { \, K_{s} = K_{s,2} } \hfill \\ \end{array} } \right. $$

## Finite element calculation model of the GRD under MSD

Finite element model analysis is an important means to study GRD laws under MSD. ANSYS is a large-scale and general finite element analysis software. It includes the SOLID65 element specially designed for concrete materials, which shows strong practicality in concrete structures. APDL (ANSYS Parametric Design Language) can easily add spring elements to simulate the interaction between the shaft and SRSM. Therefore, ANSYS is used to establish a simplified finite element model through the spring element.

### Modelling

**Figure 4:**
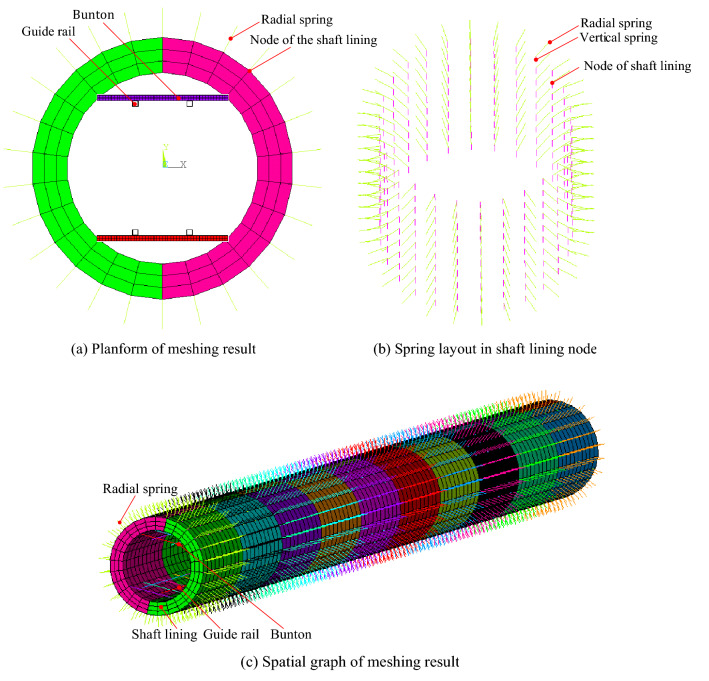
Finite element model based on spring element of SRSM under MSD.

Based on the geometric parameters of Zhangshuanglou auxiliary shaft, a finite element model is established for GRD analysis under MSD, as shown in Fig. 4, the height is 263 m, the outer radius is 4.45 m, the interval between the buntons is 4 m, and the length of the GR is 12 m and the gap between them is 4 mm. In order to avoid the influence of the combination of rock soil mass around the shaft, the topsoil is alternately arranged with sandy clay and clay, and the bedrock is sandstone and fine siltstone. The height of each layer is 10 m, and the total height of the topsoil is 60 m. GR and bunton uses the same material. It is assumed that the rock soil mass, shaft, GR and bunton are all linear elastic materials. The calculation parameters are shown in Table 1.

**Table 1:** Basic parameters of finite element model.

	Volume weight (kN/m^3^)	Elasticity modulus (MPa)	Poisson’s ratio	RC (MN/m^3^)/PCRC in horizontal (MN/m^4^)	RC (MN/m^3^) /PCRC in vertical (MN/m^4^)
Sandy clay	10.3	1	0.23	5.5	2.75
Clay	9.8	8	0.30	11	5.5
Sandstone	26	33,000	0.2	1800	900
Fine siltstone	25	20,000	0.25	1200	600
Shaft lining	23	31,500	0.2		
Guide rail	78.5	200,000	0.3		
Rebar	78.4	200,000	0.3		

The shaft lining uses an integral SOLID65 element in which the rebar is dispersed and the crushing function is turned off. GR and bunton adopt BEAM188 element, its length and width of the section are 0.2 m and the thickness is 10 mm. The deformation resistance of the SRSM acts on the shaft lining through vertical and radial spring elements, which are COMBIN39 elements and are only compressed. One end of the spring element is fixed, the other end is connected to the shaft lining node, and its stiffness coefficient is obtained according to the RC in Table 1. The bottom of the shaft adopts the fixed constraint, and the MSD is achieved by imposing a forced displacement boundary condition on the shaft lining. The direction along the bunton is defined as the *x*-axis, the direction perpendicular to the bunton is the *y*-axis, the positive direction is away from the inside of the shaft, the direction along the shaft is defined as the *z*-axis, the positive direction is vertical upward, and its origin is located at the center of the upper surface. The depth of the upper surface is 0 m.

### Model validation

Due to the restriction of field production condition, as well as the randomness and uncertainty of the mining shaft deformation type and time, the field test condition is difficult to meet. The huge size of the shaft leads to a large similarity constant in the scaled model test, and it causes the small cross-sectional dimensions of the GR and bunton in the scaled model, which cannot be fabricated and installed in the scale model. Based on the above reasons, the effectiveness of the spring element to simplify the interaction between the shaft and SRSM is verified by the calculation results of the solid element.

#### Model based on solid element

**Figure 5:**
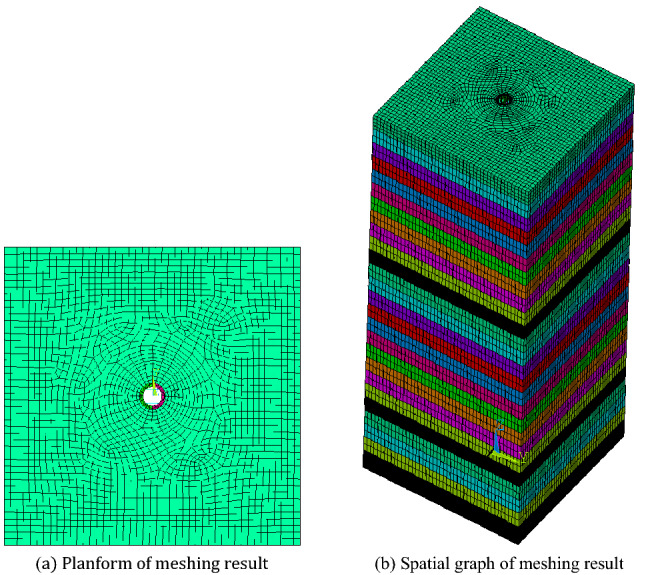
Finite element model based on solid element of SRSM.

The finite element model based on the solid element of the SRSM is shown in Fig. 5, and its size is 100 × 100 × 263 m. The surrounding interface of the SRSM is constrained by the normal displacement, and the bottom surface is constrained by the fixed. Other calculation parameters are the same as the spring element calculation model.

**Figure 6:**
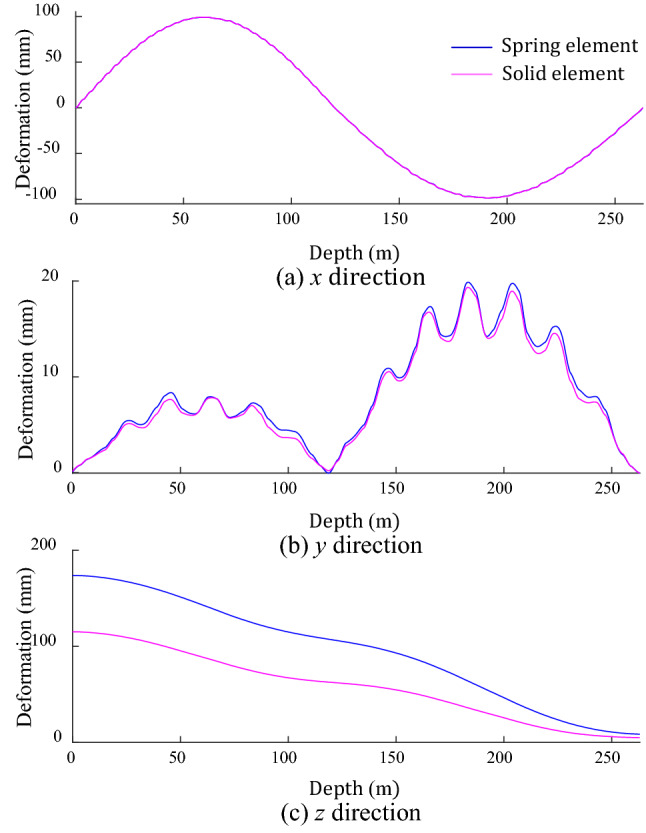
GRD under different calculation models.

Owing to the symmetry of the applied load and GR layout, the deformation laws of the GR in different directions are symmetrical, so the single-sided GR in the negative direction of *x* and *y* axes is selected as the research object. The same cosine displacement load in the *x*-direction is applied to the two models respectively, and the calculation result of the GRD is shown in Fig. 6. The deformation basically coincides in the *x* and *y* direction, and the deformation law is the same in the *z* direction, only the amplitude is different. This result indicates the accuracy of the simplified finite element model based on spring element.

The model based on spring element does not need to establish a full-size SRSM, and the calculation scale is greatly reduced compared with the solid element, which greatly reduces the requirements for computer performance and greatly improves the calculation efficiency, as shown in Table 2. The number of grids is reduced by 16.5 times and the computation time is reduced by 9.7 times. In addition, there is no need to add contact between the shaft and SRSM, which simplifies the calculation steps and improves the success rate of simulation. The workstation parameters used in the calculation are shown in Table 3.

**Table 2:** Scale and efficiency of finite element calculation model.

Model	Number of grids	Computation time(min)
Solid element	614,475	831
Spring element	37,190	85

**Table 3:** Workstation parameters.

CPU	Number of cores	Main frequency (GHz)	RAM (GB)	Cache (MB)
Intel Xeon E5-2678 v3 (two)	24	2.5	64	67.5

#### Experimental model

**Figure 7:**
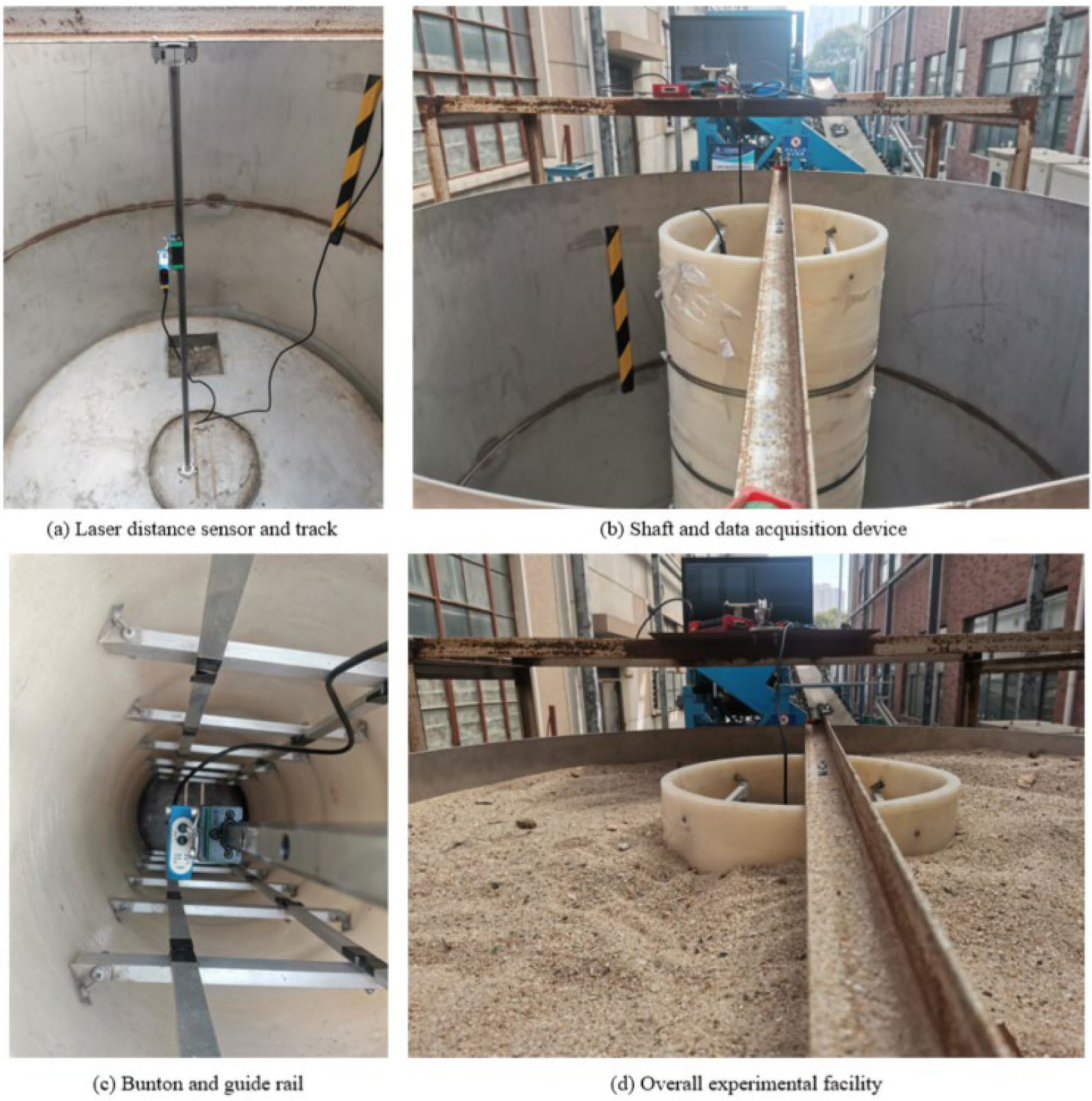
Shaft experimental model.

The shaft experimental model is shown in Fig. 7. The laser distance sensor PT5070F is mounted on a track with a slider that can be move back and forth along it to measure the GRD at different position. The deformation data is transmitted to the LabVIEW data acquisition platform through the LabJackT4 data acquisition card to realize the data recording and preprocessing. The shaft is made of rubber, the SRSM is replaced by fine sand, and the bunton and GR are made of aluminum alloy. Its geometric parameters are shown in Table 4. Due to the size of shaft lining and the arrangement of bunton and GR, this experiment can verify the GRD in the upper part 20 m range of the finite element model. The experimental and spring element model of the shaft have the same deformation type, and the GRD is compared. Since the above models do not follow the similarity theorem, only qualitative comparison can be made. Some fine sand at the bottom of one side of the shaft experiment model is removed, and the movement of the upper fine sand drives the shaft to produce inclined deformation. Meanwhile, the inclined displacement load is added to the spring element model within the range of 20 m, and the GRD calculation results were compared.

**Table 4:** Geometric parameters of shaft experimental model.

Shaft height (mm)	Shaft inner diameter (mm)	Shaft thickness (mm)	Cross-sectional area (${\text{mm}}^{2}$)
1000	162.5	12	400

**Figure 8:**
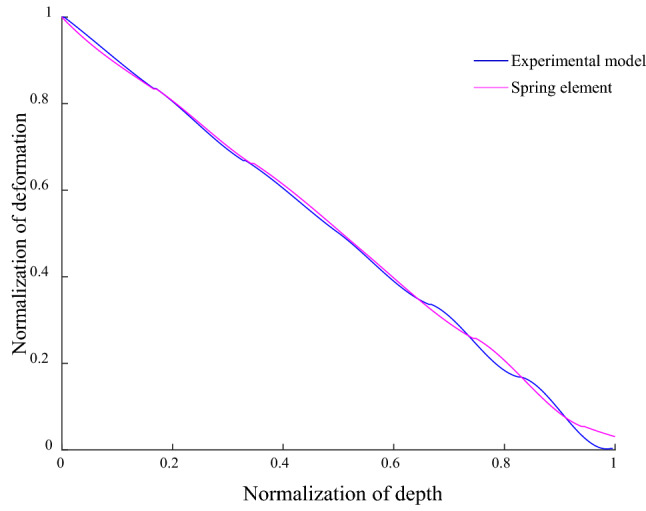
Guide rail deformation of spring element and experimental model.

In Fig. 6a, the GRD in the horizontal direction has a good tracking effect on the shaft deformation, and the GRD in this direction and its corresponding depth are normalized, as shown in Fig. 8. The wave deformation at the bottom of the two models is opposite, and the deformation in experiment is smaller than that in the spring element model, mainly due to the different constraints at the bottom of the shaft. The shaft of the experimental model is fixed on the stainless steel vessel by bolts, which is approximately fixed constraint, while the simulation model only applies displacement load to the upper local shaft, and the deformation range extends downward to a certain extent, resulting in the increase of the deformation value of the lower guide rail. However, the overall deformation trend of the spring element model is consistent with the experimental model. The guide rail deformation decreases with the increase of depth and is accompanied by the wave deformation. The wave deformation amplitude increases with the increase of depth. It is further proved that the spring element model is effective method to analyze the GRD under MSD.

## Finite element analysis result of the GRD under MSD

MSD not only causes GRD, but also easily leads to the disconnection between the shaft, bunton and GR. The GRD characteristics are analyzed under the normal connection between them, the disconnection of the shaft from the bunton, and the disconnection of the bunton from the GR. The instability of the hoisting conveyance is mainly caused by the horizontal deformation of the GR. It can be seen from Fig. 6 that the horizontal deformation of the guide rail in the same direction as the shaft deformation has a good follow-up to the shaft deformation, is highly correlated with the shaft deformation trend, and other horizontal directions are relatively low, so the main focus is on the deformation characteristic and law of the GR corresponding to the shaft deformation in the horizontal direction. Owing to the action mechanism of vertical compression deformation of shaft is different from other deformation types, it mainly focuses on inclination, bending, dislocation and horizontal section deformation of the shaft.

### Guide rail deformation under the inclination deformation of the shaft

**Figure 9:**
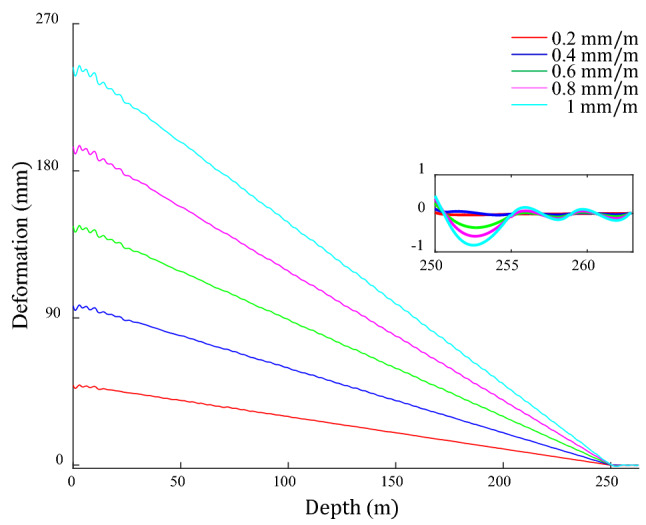
GRD in *x*-direction under inclination.

Starting from the position of − 250 m, the shaft is inclined in the positive direction of the* x*-axis, and the inclination rates are 0.2, 0.4, 0.6, 0.8 and 1 mm/m, respectively. The GRD corresponding to the *x*-direction is shown in Fig. 9. It can be found that the GRD is roughly linear and basically consistent with the shaft deformation. Its maximum deformation values are 49.1, 97.9, 146.5, 195.5 and 244.2 mm, and it is reduced to a certain extent compared with the shaft inclination deformation. The GR within a certain depth from the upper surface of the shaft has both linear and fluctuating deformation. The period of the fluctuation deformation is 4 m, which is the interval between adjacent buntons, and its amplitude and deformation range increase with the aggravation of the inclination deformation. The GR below the starting position of the inclination deformation only produces fluctuation deformation with a period of 4 m, and the amplitude increases with the aggravation of the inclination deformation.

The lower GR has a stretching action on the upper part, but the support action of the bunton on the GR hinders the stretching, which leads to the reverse deformation of the GR with the bunton as the center, forming the fluctuation deformation in the upper part. The GR above the starting position of the inclined deformation makes the lower part tend to incline, and the constraint of the bunton on the GR restricts this deformation, resulting in the fluctuation deformation of the lower part. With the aggravation of the inclination deformation, the GRD basically increases linearly, and the amplitude and range of the fluctuation deformation increase, which has a good characterization relationship for the type and degree of the inclination deformation of the shaft.

**Figure 10:**
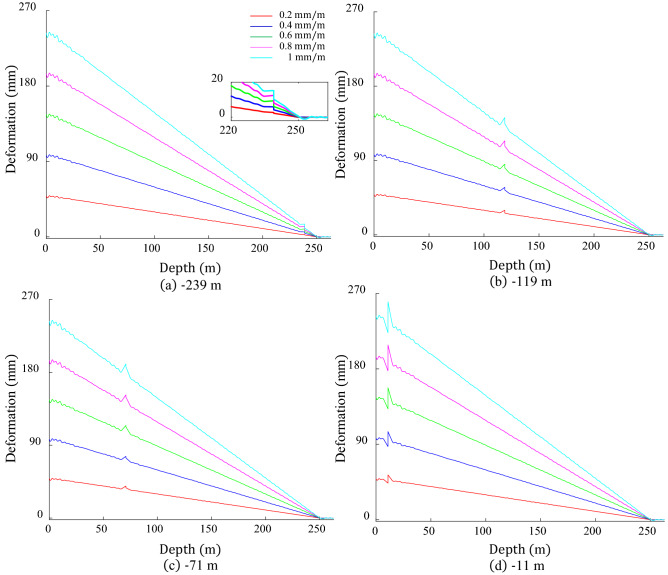
GRD in *x*-direction under inclination when bunton is disconnected from GR.

To make the disconnection representative between bunton, GR and shaft under inclined deformation, the disconnection positions are selected at the upper, middle and lower parts of the shaft, which are − 239, − 119, − 71 and − 11 m respectively. Figure 10 shows the *x*-direction deformation of the GR under inclined deformation when the bunton is disconnected from the GR. In Fig. 10a, the deformation is basically the same as the normal connection except near the disconnection position. At the disconnected position − 239 m, it is stepped with heights of 0.4, 1.8, 2.3, 4.3 and 4.9 mm, respectively. It increases with the aggravation of the inclination deformation, and the change rate first increases and then decreases. The disconnected position is affected by the inclined deformation of the shaft, and the accumulated length of the GR below it is relatively small, resulting in a relatively small cumulative deformation energy of the GR near the lower part of the disconnection position, but the deformation energy near the upper part of the disconnection position is much larger than that of the lower part. When the bunton is disconnected from the GR, the deformation energy is released, so that the deformation of the GR in the upper part of the disconnection position is larger than that in the lower part, forming a stepped shape.

The deformation law near the disconnected position at − 119 and − 71 m is basically the same, as shown in Fig. 10b,c, with a sawtooth shape, the height increases with the aggravation of inclined deformation, and its minimum value is 3.1 mm and the maximum value is 9.4 mm. Since the disconnection position is located in the middle of the inclination deformation of the shaft, the difference in the accumulated deformation energy near the upper and lower part of the disconnection position is relatively small, and the deformation caused by the release of the deformation energy is basically the same, so it has a sawtooth shape. When the bunton and GR are disconnected at − 11 m as shown in Fig. 10d, the GR on both sides of the disconnected position is deformed in reverse, showing a two-way sawtooth. The peak-to-peak value of the two-way sawtooth increases with the aggravation of the inclined deformation, and the minimum value is 9.8 mm and the maximum value is 36.5 mm, which far exceeds the allowable value of the GRD stipulated by the coal mine safety regulations. The stretching of the upper GR to the lower causes the upper bunton to be greatly stretched downward, induces the bunton to bend downward. When the upper bunton is disconnected from the GR, the bending deformation of the bunton at the disconnected position is greatly released, and the deformation at other positions is reduced to a certain extent, but the curvature is still large. Under the action of bending deformation, GR deforms to the side with smaller curvature, that is, the negative *x*-axis direction, forming a two-way sawtooth. Through the above analysis, it is found that the deformation laws of GR corresponding to different disconnected position are distinct. The bottom is stepped, the middle is sawtooth, and the upper part is two-way sawtooth. As the inclination deformation increases, its deformation amplitude augments. The disconnection position near ground has the greater impact on the GRD and is easy to cause hoisting accidents.

**Figure 11:**
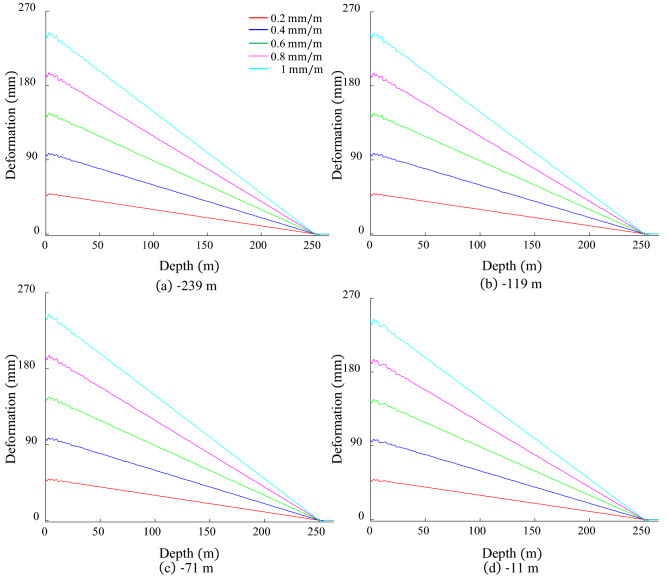
GRD in *x*-direction under inclination when bunton is disconnected from Shaft.

When the bunton is disconnected from the shaft at different positions, the *x*-direction deformation of the GR under inclination deformation is shown in Fig. 11. The GRD law is basically unchanged at the disconnected position of − 239, − 119 and − 71 m, and the overall GRD law is fundamentally the same as that in the normal connection state. The period becomes 8 m at − 11 m, as shown in Fig. 11d, and it is twice the distance between adjacent buntons, but the amplitude basically unchanged. The disconnection between the shaft and the bunton loses its supporting effect on the bunton, the constraining effect of the disconnected bunton on the GR is invalid, and the GR deforms under the action of the interval bunton, which finally causes the period of the GRD to change at this position. Since the supporting effect of the upper bunton on the GR is much stronger than that of the middle and lower parts, the upper disconnection position easily affects the guide deformation. The disconnection of the bunton and shaft has less influence on the GRD than the separation of the bunton and GR, hence the attention should be paid to the connection state of the bunton and GR under the inclined deformation of the shaft.

### Guide rail deformation under the bending deformation of the shaft

**Figure 12:**
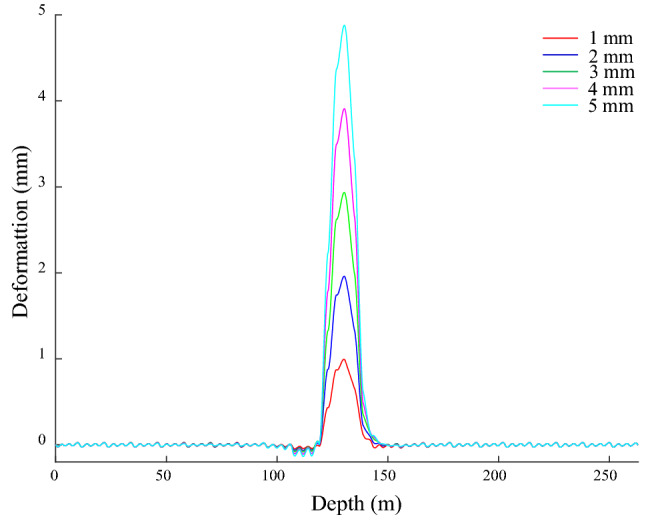
GRD in *x*-direction under bending.

The − 140 to − 120 m segment of the shaft acts on the given of *x*-direction bending deformation, which is cosine type, the amplitudes are 1, 2, 3, 4 and 5 mm in turn. The GRD in the *x*-direction is shown in Fig. 12, there is approximately symmetrical bending from − 146 to − 119 m, and the deformation ranges are (− 143.1, − 119.1), (− 147.1, − 119.1), (− 147.9, − 119.1), (− 148.7, − 119.1) and (− 149.8, − 119.1) m, which are larger than the bending deformation range of the shaft. The maximum value of GRD is 0.99, 1.96, 2.93, 3.90, and 4.87 mm, the corresponding position is − 130.2 m, and it is maximum deflection position of the shaft. There are differences in the GRD law on both sides of the maximum deflection, the right side is a smooth curve, and the left side is an inclined stepped curve. The inclined stepped curve is mainly due to the asymmetry of the bunton with respect to the maximum deflection position of the shaft. The bunton closest to the maximum deflection position of the shaft is located on its lower side, the maximum deflection of GR appears near the bunton under its action, and the distance relationship causes the above action on the lower side of the maximum deflection of the shaft to be much greater than the upper side, so the attenuation rate of the guide deformation is relatively slowly above the maximum deflection position of the shaft. Nevertheless, the effect of the farther upper adjacent bunton on it further increases its attenuation rate and it becomes the stepped shape. There is a corresponding relationship between the GRD and the bending deformation of the shaft. As the amplitude of the bending deformation of the shaft increases, the amplitude and range of the bending deformation of the GR enlarge, and the deformation law is the same. It indicates the sensitivity and reliability of the GRD to characterize the bending deformation of shaft.

GRD appears periodic weak fluctuation outside a certain distance from the shaft bending region, its period is 4 m, and its amplitude basically does not change with shaft bending deformation, which indicates that its influence on the GR is limited. From − 119 to − 106 m, the deformation generally exhibits an inverted arch, and its amplitude is larger than other positions of the fluctuation deformation and it increases with the aggravation of the shaft bending deformation. The bunton’s constraint on the bending deformation of the GR makes it subject to a force in the negative *x*-direction, and drives the GR to move along the negative *x*-direction within a certain range, and finally the inverted arch occurs. The fluctuation is mainly caused by the constraint effect of bunton on the GR. The action of gravity leads the GR to deform in the vertical direction, and the constraints limit this deformation, which makes the upper half of the GR between adjacent buntons deforms along the negative *x*-direction, the lower half deforms along the positive *x*-direction, and the joint has the smallest deformation and is the starting point of periodic fluctuation. The maximum amplitude of the above fluctuations is only 0.02 mm, the verticality and straightness meet the requirements of the GR installation specification, and it is generally straight, which further indicates the rationality of the initial connection parameters of the bunton, GR and shaft.

**Figure 13:**
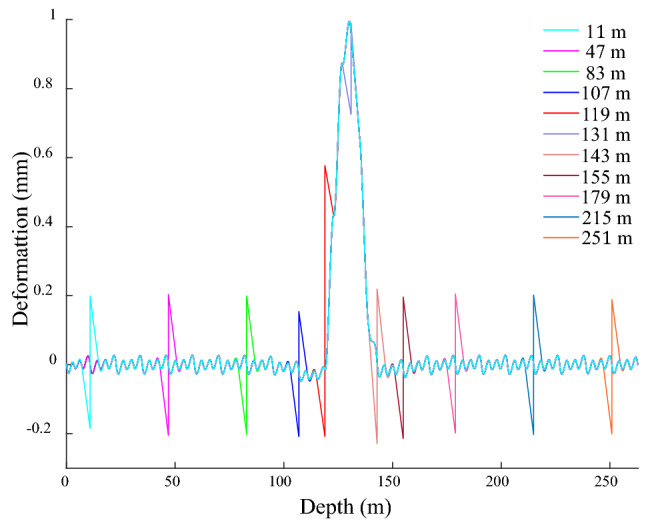
GRD in *x*-direction under bending when bunton is disconnected from GR.

The disconnection positions are − 11, − 47, − 83, − 107, − 119, − 131, − 143, − 155, − 179, − 215 and − 251 m in sequence, and are all over the bending position and both sides of the shaft. Owing to the same of GRD law under different degrees of shaft bending deformation, it is studied that the shaft bending deformation amplitude of 1 mm on disconnection. Figure 13 shows the GRD law when the bunton is disconnected from the GR under bending action. The GR at the disconnected position deforms in opposite direction, and its amplitude is 0.38 mm outside the range of shaft bending deformation, which is about 7 times the amplitude of the fluctuation deformation. The maximum amplitude occurs at − 119 m, and it is about 14 times the amplitude. This disconnection position is located at the junction of the shaft bending deformation, and the bunton below it is situated in the action area of the shaft bending deformation, so the GR below it is subjected to the above action, resulting in its abnormally prominent amplitude. The disconnected position at − 131 m is located in the middle of the bending deformation area of the shaft, the GR above it produces the reverse deformation and its amplitude is 5 times that of the fluctuation deformation, and the deformation below it is basically the same as that of the normal connection. Due to the bending deformation of the shaft, the end of the adjacent bunton at the disconnected position is bend towards the inside of the shaft, it causes the bunton near the disconnection position is bent downward, the lower GR is bent in the positive *x*-direction, and the upper GR is deformed in the opposite direction, which eventually leads to the GR occurs opposite deformation at the disconnected position between the bunton and GR.

**Figure 14:**
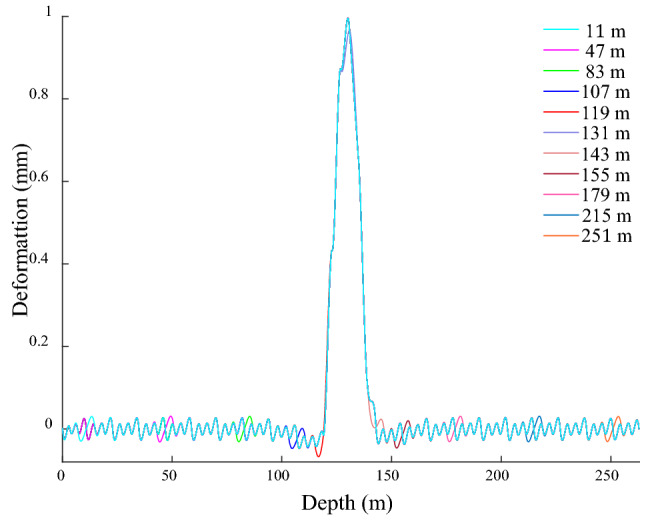
GRD in *x*-direction under bending when bunton is disconnected from shaft.

When the bunton is disconnected from the shaft under the action of the shaft bending deformation, the GRD is shown in Fig. 14. When the disconnection is outside the shaft bending deformation, the period of the fluctuation deformation changes at the disconnection position and becomes 8 m, and the amplitude basically does not change. When the disconnected position is located at − 119 m at the upper junction of the shaft bending deformation, the GRD law on the lower side of the disconnected position is basically the same as the normal connection. The deformation on the upper side of the disconnected position extends in the negative *x*-direction, which is mainly due to the bending effect of the lower GR in the positive *x*-direction and the reverse restraint effect of the upper GR in the disconnected position. When the disconnected position is located at − 143 m at the lower junction, the deformation decreases on the upper side near the disconnected position, and it increases on the lower side. The GR above this disconnected position is located in the bending deformation area of the shaft, it acts on the GR with an active force in the positive *x*-direction, thereby producing a pulling effect on the lower GR in the positive *x*-direction. The bunton at this disconnection position is located outside the bending deformation area of the shaft. When it is normal connection, it produces a negative *x*-direction constraint on the GR to slow down its attenuation rate of the bending deformation, the above restraint is eliminated under the disconnection state cause an increase in the attenuation rate of the GR bending deformation, which induces a diminution in the upper GRD near the disconnected position. and the bending deformation range of the GR and the deformation value below it increases.

When the disconnected position is − 131 m in the bending deformation area of the shaft, the deformation law near the disconnected position is basically the same as the normal connection, and the period and amplitude do not change significantly. When the disconnected bunton is located in the bending deformation area of the shaft, and the adjacent bunton has a strong bending deformation force on the GR at the same time, so even if a single bunton is disconnected from the GR, its force condition is basically unchanged, resulting in basically no change in its deformation law and amplitude. When the disconnected bunton is outside the bending deformation range of the shaft, the GR deformation period at the disconnected position changes, but the amplitude does not change. When the disconnected bunton is located at the junction of the shaft bending deformation, the deformation and period at the disconnected position change at the same time. The disconnection between the bunton and shaft under the bending deformation action has little influence on the deformation law of the GR, and the connection between the bunton and the GR should be focused on.

### Guide rail deformation under the dislocation deformation of the shaft

**Figure 15:**
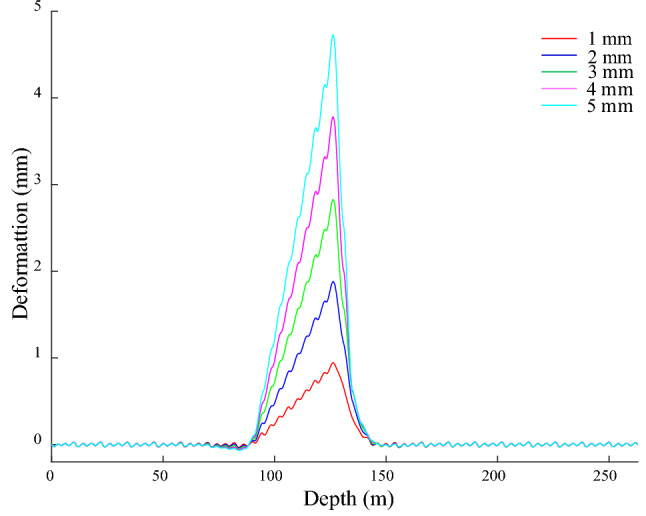
GRD in *x*-direction under dislocation.

For a given shaft dislocation deformation in the *x*-direction from − 130 to − 90 m, the amplitude is 1, 2, 3, 4 and 5 mm at dislocation position of − 130 m and becomes 0 mm at the − 90 m, and the attenuation rate is 0.025, 0.05, 0.075, 0.010 and 0.0125 mm/m. The GRD under dislocation action is shown in Fig. 15. The maximum deformation of the GR is 0.94, 1.88, 2.82, 3.78 and 4.72 mm, and its position is − 126.2 m, which is higher than the dislocation position of the shaft, but its amplitude is basically unchanged compared with the dislocation amplitude of the shaft. The distance between the adjacent bunton makes it difficult to coincide with the position of the dislocation deformation of the shaft, and the dislocation deformation effect is transmitted to the GR through the bunton, so this effect causes the maximum deformation of the GR to occur near the bunton, and its position is moved up than the dislocation position of the shaft. The influence range of the dislocation deformation on the GRD is (− 143.5, − 88.3), (− 143.9, − 88.3), (− 146.7, − 88.3), (146.9, − 88.3) and (− 146.9, − 88.3) m, which is much larger than the action scope of the shaft dislocation deformation. The shaft above the dislocation interface produces a stretching effect on the lower part, its lower part moves to the positive *x*-direction and expands the shaft deformation range, but the resistance from the SRSM to shaft restricts the transmission range of the above effect, which induces the deformation range of the GR is much larger than the action range of the shaft dislocation deformation. There are differences in the deformation law of the GR on both sides of the maximum amplitude, the upper side shows the attenuation trend of inclination deformation, and its attenuation rate is basically the same as the shaft dislocation deformation, accompanied by fluctuation deformation. The lower side has a bending deformation trend, and the attenuation rate first increases and then decreases as it moves away the maximum amplitude, which is mainly caused by the follow-up deformation of the shaft below the dislocation interface. The GRD corresponding to the shaft dislocation is a combination of inclination and bending. With the increase of the dislocation deformation, the slope of the inclined deformation of GR, the curvature of the bending deformation and the maximum amplitude increase, which are obviously different from the deformation feature of GR under shaft bending or inclination, and there is a good mapping relationship for the shaft dislocation.

**Figure 16:**
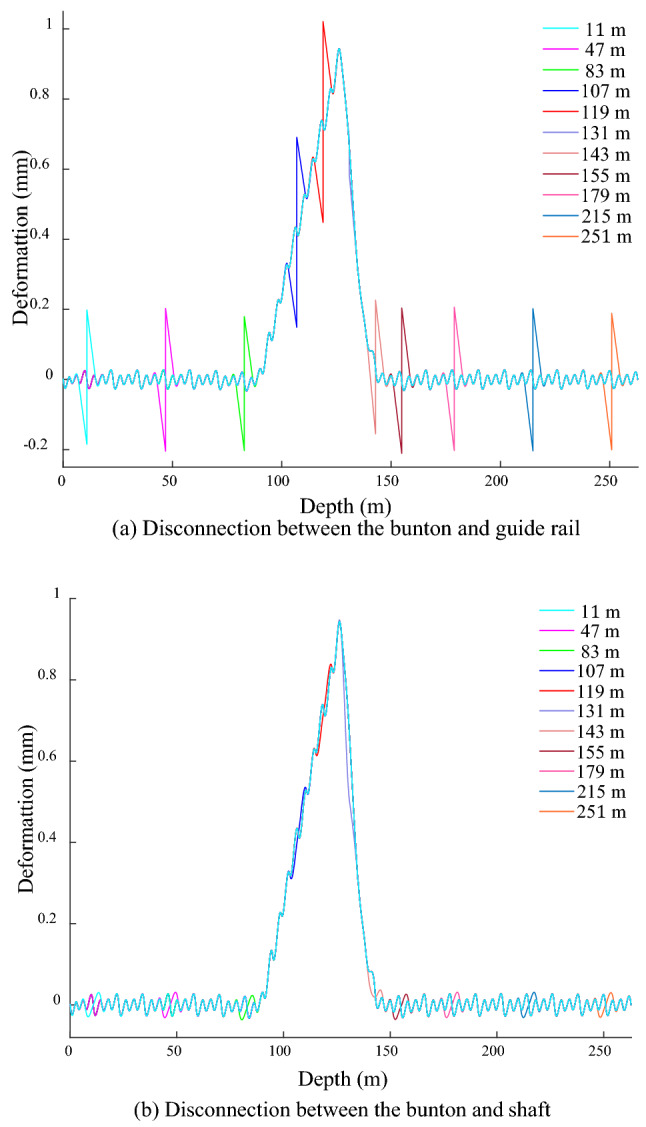
GRD in *x*-direction under dislocation when connection is abnormal.

Figure 16a shows the *x*-direction deformation of the GR when it is disconnected from the bunton under the action of the shaft dislocation, its position is − 11, − 47, − 83, − 107, − 119, − 131, − 143, − 155, − 179, − 215 and − 251 m, and the GR in the disconnected position generates the opposite deformation. The peak-to-peak value of the opposite deformation outside the shaft dislocation range is about 0.4 mm, and it is about 20 times fluctuation deformation, which is mainly caused by the release of the interaction between the GR and bunton under the action of gravity. When the disconnection between the bunton and GR is − 107 and − 119 m in the shaft dislocation area, the peak-to-peak value of the opposing deformation is 0.53 and 0.57 mm, respectively, and its value increases less compared to outside of the shaft dislocation deformation region. Moreover, the peak-to-peak value at the junction of the shaft bending deformation is basically the same as that outside the shaft dislocation area. When the bunton is disconnected from the shaft at different positions, the GRD is shown in Fig. 16b. The deformation decreases near the upper side of the disconnected position of − 143 m, the lower side augments, and its deformation mechanism is the same as at this position under the action of the bending deformation of the shaft, hence the explanation is not repeated here. Other disconnected position is basically unchanged in amplitude, and only the period becomes twice the fluctuation deformation. The deformation law is basically the same as the normal connection except the above disconnection position, hence the influence of shaft dislocation deformation on the GR has no obvious attenuation due to the disconnection between the bunton and GR.

### Guide rail deformation under the horizontal section change of the shaft

**Figure 17:**
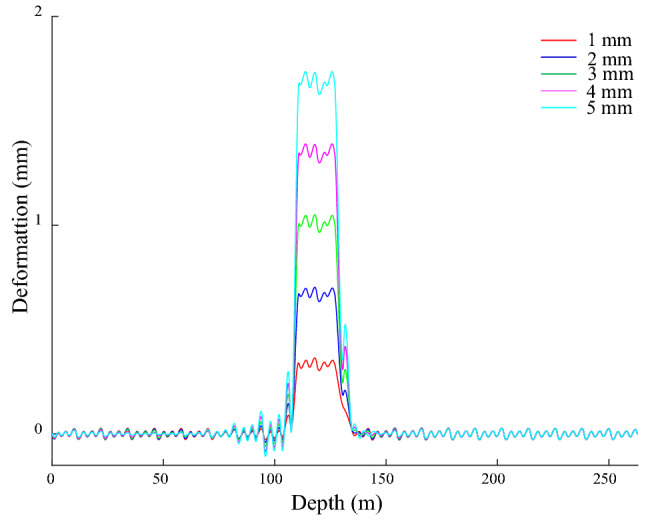
GRD in *x*-direction under horizontal section change.

The shaft of the − 130 to − 110 m segment imposes a given displacement in the *x*-direction so that its cross-section is approximately elliptical, the short axis is the *x*-axis and the long axis is the *y*-axis, and the amplitude occurs at the intersection of the outer wall of the shaft and *x*-axis. It attenuates uniformly on both sides along the *y*-axis and becomes 0 mm at the intersection of the shaft and *y*-axis, and the attenuation curve is cosine. Figure 17 shows the GRD when the amplitudes of the horizontal section change of the shaft are 1, 2, 3, 4 and 5 mm in turn. The GRD from − 134.9 to − 107.4 m is mainly affected by the horizontal section change, and the interval is basically unchanged with the aggravation of the horizontal section change. The deformation is generally trapezoidal, the middle section is a horizontal straight line accompanied by fluctuation deformation, and the transition area decays rapidly, generally an inclined straight line. With the increase of the deformation amplitude of the horizontal section change, the GRD from − 134.9 to − 130.5 m shows the local arching phenomenon, which becomes more severe with the aggravation of the above deformation. The above phenomenon occurs near the junction of the action and influence zone of the horizontal section change. The bunton in action area follows the shaft to deform in the positive *x*-direction, and the GRD in the affected aera is less affected by the horizontal section change of shaft and bunton in affected area has a strong constraining effect on the guide deformation in the positive *x*-direction, and the GR in action area exists a strong stretching effect on GR in this position, which induces the local arching phenomenon.

The amplitudes of the GRD are 0.33, 0.70, 1.04, 1.38, and 1.73 mm corresponding to each horizontal section change, and it is a large attenuation relative to the shaft deformation. The fluctuation deformation amplitude from − 107.4 to − 73.1 m augments with the aggravation of the horizontal section change, its amplitude is no obvious change rule from − 73.1 to − 46.9 m, and the amplitude of − 46.9 to 0 m decreases. The above phenomenon is mainly caused by the strong constraint effect of bunton of the transition zone on the GR, and the strong stretching effect of the lower GR on the upper part. The deformation law of the GR is basically the same under the different amplitude of horizontal section change of the shaft, but there are obvious differences in the amplitude, which exists a good corresponding relationship between the horizontal section change of the shaft and the deformation law of the GR.

**Figure 18:**
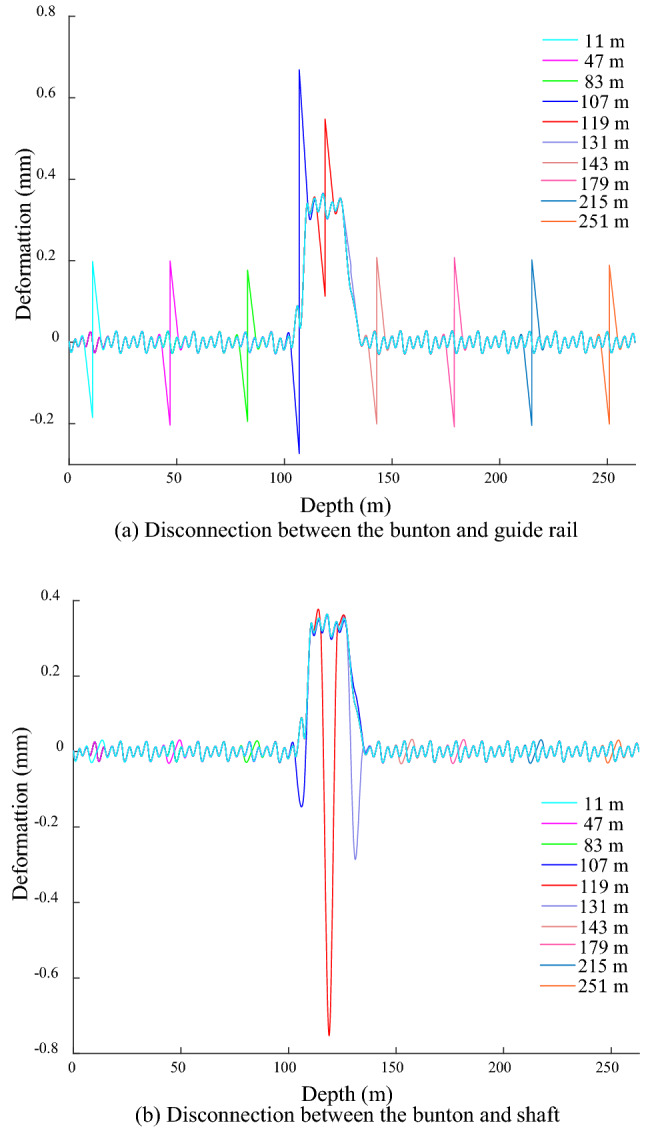
GRD in *x*-direction under horizontal section change when connection is abnormal.

When the bunton is disconnected from GR, its position is − 11, − 47, − 83, − 107, − 119, − 131, − 143, − 179, − 215 and − 251 m, respectively, and the GRD is shown in Fig. 18a under horizontal section change. It generates the opposite deformation at the disconnected position, and its maximum peak to peak occurs in the transition zone of the horizontal section change at − 107 m and its value is 0.94 mm. The disconnected bunton in the transition area locates in the outside of the shaft deformation and has little deformation effect on the GR, and the bunton below the transition area locates in the shaft deformation area and follows the shaft deformation to drive the GRD, which causes a large difference in force between adjacent buntons, and the maximum peak-to-peak value appears. In the horizontal section change area of the shaft at − 119 m, the peak-to-peak value is 0.43 mm, and its value is basically the same as the disconnection position outside action area of the horizontal section change of the shaft. The disconnection between the bunton and GR in the transition area has the greatest influence on the GRD, which can easily lead to the abnormal deformation.

Figure 18b is the GRD corresponding the disconnection between the bunton and shaft under the horizontal section change of the shaft. The disconnection outside the action and transition area of the shaft deformation only changes the fluctuation deformation period near the disconnection position and its value is 8 m, and the amplitude is basically unchanged. In the shaft deformation action area, the maximum peak-to-peak value is 1.12 mm at disconnection position of − 119 m, and the peak-to-peak value at − 107 and − 131 m in the upper and lower transition areas are 0.57 and 0.62 mm respectively. The buntons on both sides of the disconnected position are located in the shaft deformation action area, are subjected to the pulling effect of the shaft in the positive *x*-direction, but the bunton at the disconnection position lacks the above effect, which eventually leads to local inverted arch near the position. The deformation principle of the GR at the disconnection position of the transition zone is the same as the above, except that one side of the bunton is located in the action zone, and the other side is located in the transition zone. However, the effect in transition zone is relatively weak, resulting in a relative lower peak-to-peak value. When the bunton is disconnected from the GR or shaft, the horizontal section change has a great influence on the GRD, so attention should be paid to the connection state.

## Conclusion

In order to study the GRD law under the MSD, the calculation formula of the spring stiffness coefficient that simplifies the interaction between the shaft and the SRSM is deduced, and the finite element calculation model based on the spring element is established for the analysis of the deformation law of the GR under MSD, and the following conclusions are obtained:

1. The finite element calculation model based on the spring element can better simulate the interaction between the shaft and SRSM. Compared with the solid element model, the calculation steps are simple, and the calculation scale is greatly reduced, which greatly improves the efficiency of simulation analysis.
2. There are obvious differences in the deformation law of the GR under different types of MSD, it is highly correlated with the shaft deformation trend, and has a strong ability to characterize the shaft deformation. Under the same MSD type, the GRD increases significantly with the aggravation of the shaft deformation, the GRD range is expanded in contrast with the shaft deformation, and the amplitude is attenuated to a certain extent. The deformation range under the dislocation action has the strongest expansion ability, and the amplitude has the fastest decay rate under the horizontal section change.
3. When the bunton is disconnected from the GR under the MSD, the GRD at the disconnected position is basically two-way sawtooth, and its peak-to-peak value is different due to the disconnection position and the MSD type. When the bunton is disconnected from the shaft, the period of the GRD is doubled at the disconnected position, it basically has no influence on the amplitude except for the horizontal section change. Compared with the disconnection of the bunton and GR, it has less impact, and attention should be paid to the connection state between the bunton and GR.
4. The paper only studies the deformation law of the GR under the single MSD type. With the continuous deepening of the shaft and the increasingly complex geological conditions, the deformation law of the GR under the composite MSD type should be further studied.

## Data Availability

The datasets used and/or analyzed during the current study available from the corresponding author on reasonable request.
